# Sociodemographic, Lifestyle, Environmental and Pregnancy-Related Determinants of Dietary Patterns during Pregnancy

**DOI:** 10.3390/ijerph16050754

**Published:** 2019-03-02

**Authors:** Ewelina Wesołowska, Agnieszka Jankowska, Elżbieta Trafalska, Paweł Kałużny, Mariusz Grzesiak, Jolanta Dominowska, Wojciech Hanke, Gemma Calamandrei, Kinga Polańska

**Affiliations:** 1 Department of Environmental Epidemiology, Nofer Institute of Occupational Medicine, 91-348 Lodz, Poland; ewelina.wesolowska@imp.lodz.pl (E.W.); agnieszka.jankowska@imp.lodz.pl (A.J.); pawel.kaluzny@imp.lodz.pl (P.K.); wojciech.hanke@imp.lodz.pl (W.H.); 2 Department of Hygiene and Epidemiology, Medical University of Lodz, 90-647 Lodz, Poland; elzbieta.trafalska@umed.lodz.pl; 3 Obstetrics, Perinatology and Gynecology Department, Polish Mother’s Memorial Hospital Research Institute, 93-338 Lodz, Poland; mariusz.grzesiak@gmail.com; 4 Department of Teaching Midwifery, Medical University of Lodz, 90-419 Lodz, Poland; jolad48@wp.pl; 5 Centre for Behavioral Sciences and Mental Health, National Institute of Health, I-00161 Rome, Italy; gemma.calamandrei@iss.it

**Keywords:** dietary patterns, pregnancy, sociodemographic, lifestyle, environmental, pregnancy-related factors, exploratory factor analysis

## Abstract

*Background:* Characterization of dietary patterns represents a valid and meaningful measure of overall diet quality and nutrient intake. The study aims at evaluating the sociodemographic, lifestyle, environmental, and pregnancy-related determinants of maternal dietary patterns during pregnancy. *Methods:* The analysis was conducted on a group of pregnant women from the Polish Mother and Child Cohort (REPRO_PL). During the second trimester of pregnancy, 1306 women filled in a modified version of the validated food frequency questionnaire (FFQ). Dietary patterns were estimated using an exploratory factor analysis. Potential dietary determinants were assessed via a questionnaire or biomarker measurements (saliva cotinine level). *Results*: Two dietary patterns were identified. The Prudent dietary pattern was characterized by high consumption of fruit, vegetables, legumes, whole grains, poultry, and low-fat dairy products, while the Western dietary pattern included high intake of refined grains, processed meat, potatoes, and very low intake of whole grains. Clear sociodemographic, environmental, lifestyle, and pregnancy-related determinants of diet quality were established. Older age (β = 0.2; *p* < 0.01), higher educational level (β = 0.3; *p* < 0.01), and socioeconomic status (SES) (β = 0.2; *p* < 0.01), overweight/obesity before (β = 0.3; *p* < 0.01), as well as physical activity during pregnancy (β = 0.2; *p* = 0.01) were positive determinants of a healthier diet (increasing Prudent–Western difference in dietary pattern scores). On the other hand, parity (β = −0.2; *p* = 0.04) and living in small cities (β = −0.3; *p* = 0.02) were significantly related to a rather Western dietary pattern. *Conclusions:* The current study presents evidence on specific factors influencing dietary patterns. They need to be accounted for in educational programs and interventions focused on healthy diet recommendations during pregnancy.

## 1. Introduction

Diet during pregnancy is recognized as one of the environmental factors that can have an impact on maternal health, influencing fetus and children’s development and health across the whole life course. The existing research, although still not all conclusive, has primarily evaluated an association between the nutritional status during pregnancy and birth outcomes as well as offspring adiposity, cardiometabolic, neurodevelopmental, and respiratory health with epigenetic alterations proposed to be a crucial underlying mechanism [1,2,3,4,5,6,7,8].

While many analyses in this field have so far focused on individual nutrients, recent epidemiological studies have underlined the importance of assessing the impact of the overall diet quality on health, stressing the concept of dietary patterns. This approach has conceptual and methodological advantages over studies based on a single dietary product. Firstly, it takes into account complex interactions between food components [9,10]. As pointed out by Hu (2002), some nutrients are highly correlated, so it might be difficult to establish their separate effect [9]. It is also possible that the effect of an individual food component may not be big enough to be detected. In addition, the analysis of many individual food items can produce statistically significant associations by chance, or it can be confounded by correlated dietary components. Finally, the dietary pattern approach allows for trends and cultural aspect analyses. It is more comprehensive to the population and stakeholders and thus, it is more useful for development of clear public health guidelines. 

Identification of factors that influence dietary choices is crucial for the assessment of population needs and development of effective public health messages and interventions. A recently published systematic review, based on the results from 12 studies, has indicated that a diet in pregnancy is patterned along a social gradient and aligned with other health behaviors (with older, better educated, affluent, nonsmoking, and physically active women being more likely to follow healthier dietary patterns) [11]. Knowledge about environmental pollutants that can be present in the food, such as methyl mercury (exposure mostly through fish consumption), or endocrine-disrupting chemicals (exposure mostly through food packaging) and about psychological conditions including depression, anxiety, and sleeping problems can also contribute to dietary choices. As a conclusion from that review, the authors have recommended that more studies should be conducted, particularly assessing environmental and pregnancy-related factors as potential determinants. What is more, some country-specific differences in determinants of a healthy diet might occur. 

This study aims at evaluating not only sociodemographic factors (age, education, marital status, socioeconomic status (SES), occupational activity) but, as recommended, also environmental factors (place of residence, seasonality), lifestyle/health-related behavior (smoking, alcohol, physical activity, level of psychological stress, pre-pregnancy body mass index (BMI), weight gain during pregnancy, supplement use), and pregnancy-related determinants (parity, week of pregnancy of the first medical-care visit, pregnancy-related symptoms/complications) of maternal dietary patterns during pregnancy.

## 2. Material and Methods

### 2.1. Study Design and Population

The Polish Mother and Child Cohort (REPRO_PL) is an ongoing population-based study designed to investigate the effects of sociodemographic, environmental, lifestyle, and pregnancy-related factors on children’s development and health. The cohort was established in 2007. The study design has been presented in detail previously [12,13,14]. Briefly, the women were invited to participate in the study if they fulfilled the following inclusion criteria: Up to 12 weeks of single pregnancy, no assisted conception, no pregnancy complications, and no chronic diseases as specified in the study protocol, which means in principle not requiring special diets. The cohort comprises the following stages: Phase I—pregnancy period, phase II—child exposure and health status assessment at the age of 1 and 2, and phase III—child evaluation at the age of 7. Each stage of the study has been reviewed and approved by the Ethical Committee of the Nofer Institute of Occupational Medicine, Lodz, Poland (Decision No. 7/2007; No. 3/2008 and No. 22/2014) and respective written informed consents were obtained from the participants following the study protocol. The present analysis includes the data that have been collected within phase I of the study [12,13,14].

Following the inclusion criteria, 1763 women were recruited into the REPRO_PL cohort [12,13,14]. Information regarding their diet was provided, thanks to completing a questionnaire conducted during the 2nd trimester of pregnancy by 1508 (85.5%) women (19 women were excluded from the original group as they described themselves as vegetarian/vegan or following a specific diet). Of this group, 1306 women filled in the validated food frequency questionnaire (FFQ) and 202 women completed the 24-hour dietary recall questionnaire (24HR) (not included in the current analysis). 

### 2.2. Dietary Pattern Assessment

#### 2.2.1. Food Frequency Questionnaire

Between the 20th–24th week of pregnancy, the women filled in a modified version of the validated FFQ [15,16]. They were assisted by trained personnel [17]. FFQ assesses consumption frequency, portion size, preparation method, and additions to the dish [18]. For each food item, including single dietary product (i.e., apples, butter, potatoes) and combined food groups created based on the similarity of nutrient profiles or culinary consumption (i.e., broccoli, cabbage, cauliflower group), the respondents reported frequency of average consumption. The following answers (frequency categories) were possible: (a) never, (b) less than once per month, (c) 1–3 times per month, (d) 1–3 times per week, (e) 4–6 times per week, and (f) every day. 

#### 2.2.2. Preprocessing of the Dietary Data

In order to apply quantitative meaning to the frequency categories, the data were converted into the following numerical values (food-item scores): (a) 0 (never), (b) 0.03 (1/30; less than once per month), (c) 0.07 (2/30; 1–3 times per month), (d) 0.29 (2/7; 1–3 times per week), (e) 0.71 (5/7; 4–6 times per week), and (f) 1 (every day). 

Subsequently, the food items were grouped to 14 predefined food groups on the basis of similarity of nutrient profiles and comparable usage [18,19]. Numerical values were assigned to the food groups by (1) taking the average of all nonzero scores of individual food items in the group and (2) rounding the result upwards to the nearest numerical value from the scores assigned to the questionnaire answers. If all individual scores in the group were equal to zero, then the group score was also set to zero. 

### 2.3. Sociodemographic, Lifestyle, Environmental, and Pregnancy-Related Factors

The following sociodemographic variables were considered: maternal age (based on the date of birth), educational levels (years of completed education: ≤9, 10–12, >12), marital status (married, unmarried), occupational activity between the 8th–12th week of pregnancy (yes, no) and SES (as described by Polanska et al. 2017) [20]. Seven factors related to lifestyle/health behavior, namely: Maternal smoking status (with 10 ng/ml as a cut-off point for cotinine level in saliva being an indicator of current active smoking, as described by Polanska et al. 2016), alcohol consumption and physical activity in the 1st trimester of pregnancy (yes, no), the level of stress (based on the perceived stress scale; PSS, as described by Polanska et al. 2017), pre-pregnancy BMI (kg/m^2^), weight gain during pregnancy (difference between the latest weight before delivery and pre-pregnancy weight categorized as low, recommended, and high weight gain in relation to maternal pre-pregnancy BMI according to the existing guidelines), and supplement/vitamins use (filled in by a gynecologist or midwife), were assessed [20,21,22,23]. In addition, two environmental variables, place of residence (based on the number of inhabitants in the proximity of residence) and seasonality (as the month of completion of the FFQ: December–February, March–May, June–August, September–November) were investigated. Pregnancy-related variables covered parity, pregnancy-related symptoms and complications which appeared after inclusion into the study (including hypertension, gestational diabetes, infections), and week of pregnancy of the first medical-care visit. The sex of the child was also included in the analysis.

### 2.4. Statistical Analysis

To derive dietary patterns from the food consumption data, we adopted the exploratory factor analysis (EFA) approach described by Hu (2002) and by other studies, which focuses on the assessment of dietary patterns during pregnancy [9,10,11]. This approach originates in the observation that consumption scores of some foods are correlated and uses statistical factor analysis to express the total variance of the dietary questionnaire scores in terms of a few latent variables (factors). 

We assumed that two factors were present in the data, which was inferred from drawing the variance explained by successive principal components of the matrix of the grouped food scores (scree plot), where there was a dropdown of the variance explained by the 3rd and the following components (Figure S1). The dietary interpretation of each factor was based on inspection of the contribution of food group variables (factor loadings).

For each food group, a factor loading was calculated, representing the extent to which the food group is related to a particular factor. Factors were determined with the criterion of the minimum sum of squared residuals, followed by “varimax” or “oblimin” rotation for interpretation. Both rotations produced factors with qualitatively equivalent factor loadings, but “oblimin” rotated factors, having larger magnitude of loadings, were selected for the data presentation.

The positive loading for a food group on a factor means that the factor represents preference towards a food group, while negative value implies that factor represents avoidance (lower than average frequency of consumption) of foods in the group. The foods with the absolute values of factor loadings of ≥0.2 on a factor were considered to have an “interpretable” association with that factor and were used to describe and label each pattern [18]. 

The two factors explain in total 18% of the variation in the dietary data (Table 1). This percentage is comparable to those reported in other studies in this field [18,19,24]. The labels that have been given to dietary patterns (Prudent and Western) do not perfectly describe each underlying pattern but aid in the report and discussion of the results.

As an overall measure of the respondents’ relative tendency towards Prudent or Western dietary pattern, a new variable was used. This variable was calculated as the difference between the food group scores for the primarily Prudent and the primarily Western dietary food groups. The obtained results were categorized into three categories (cut-off points: 33.3rd and 66.6th percentiles) to rather Western (most negative values of the difference), Mixed (moderate values at the center part of the histogram distribution), and rather Prudent (most positive values of the difference).

The association of sociodemographic, lifestyle, environmental, and pregnancy-related determinants of dietary patterns in each of the three dietary categories was assessed with the Pearson’s chi squared test. 

Seasonal variation of the food groups, according to the time of the questionnaire data collection, was assessed by regressing FFQ score of each food group on the categorical variable representing current season of the year (four levels as described above). The significance of the season variable as a predictor for the food group consumption was assessed with the F test. 

Finally, the multiple linear regression analysis was applied to identify sociodemographic, lifestyle, environmental, and pregnancy-related determinants of dietary patterns during pregnancy (separately for Western dietary pattern score, Prudent dietary pattern score, and the score differences for Prudent dietary pattern and Western dietary pattern). As an alternative, a logistic regression model was also determined, with the same set of explanatory variables, where the variables were binary indicators of Western, Prudent dietary patterns, and their difference scores falling above their respective median values (the results are presented as supplementary materials). 

The facilities of base *R* system for statistical computing [25] were used for regression modeling, and the package *psych* was additionally used for the factor analysis [26]. 

## 3. Results

### 3.1. Characteristics of the Study Population

The sociodemographic, lifestyle, environmental, and pregnancy-related characteristics are presented in Table S1. More than 60% of the women were 30 years of age or younger, 80% were married, and 90% declared a medium or high SES. Fifty-two percent of the women were at their first pregnancy. A high proportion of the women had a university degree (69%). Based on the pre-pregnancy BMI, 18% of the respondents were classified as overweight or obese, and median gestational weight gain was 12.0 kg (IQR 9–15 kg). According to the cotinine level in saliva collected in the 1st trimester of pregnancy, 11% of the participants were defined as smokers. About 6% of the women indicated alcohol consumption and 69% physical activity during pregnancy. About 60% of the women declared the 1st medical-care visit at the 6th week of pregnancy or earlier, about 3% of them noticed complications, and more than 90% used supplements up to the 2nd trimester of pregnancy. 

### 3.2. Maternal Diet during Pregnancy

The frequency of consumption of the grouped food products is presented in Table S2. About 60% of the women indicated fruit and 50% vegetable consumption 4-6 times per week (everyday consumption was indicated only by 3.3% and 1.2%, respectively). Fewer than 35% of the women declared fish consumption at least once per week and 5% did not eat it at all. Whole grains were consumed 1–6 times per week by 68% of the participants, and 2.5% indicated such consumption on an everyday basis. Such percentages were higher for the consumption of refined grains. More than 50% of the women declared consumption of red meat 1–3 times per week and 17% even more frequently. The seasonality of the consumption was significant for fruit and vegetables (*p* < 0.01) (Table S3). 

Table 1 shows the factor loadings obtained from the EFA. The Prudent dietary pattern was characterized by high consumption of fruit, vegetables, legumes, whole grains, poultry, and low-fat dairy products, while the Western dietary pattern by high intake of refined grains, processed meat, potatoes, and very low intake of whole grains. 

### 3.3. Sociodemographic, Lifestyle, Environmental, and Pregnancy-Related Determinants of Dietary Patterns in Pregnancy

The Prudent dietary pattern was represented more frequently by older (*p* = 0.05), married women (*p* = 0.03), and those declaring a higher level of education (*p* < 0.01), occupational activity (*p* = 0.03), and high socioeconomic status (*p* < 0.01) (Table 2). Interestingly, in the group of women that were classified as overweight or obese (BMI ≥ 25kg/m^2^), 23% of them represented the Western and 37% the Prudent style of eating, whereas in the group with BMI within the recommended range, they were equally distributed in each dietary pattern (*p* < 0.01). The women who were classified as smokers during pregnancy reported a Western dietary pattern more frequently than the nonsmokers (*p* < 0.01). What is more, those who declared physical activity (*p* < 0.01) as well as supplement use during pregnancy (*p* = 0.04) and those who lived in bigger cities (*p* < 0.01) were likely to indicate a healthy dietary style more frequently. 

Independent sociodemographic, lifestyle, environmental, and pregnancy-related determinants of dietary pattern during pregnancy based on the multivariate linear regression model are indicated in Table 3. Only two factors showed a significant association with the Western dietary pattern. Increasing scores were evident for parity (β = 0.3; *p* < 0.01) and decreasing ones for the women with pre-pregnancy BMI ≥25kg/m^2^ (β = −0.3; *p* < 0.01). Prudent dietary pattern was significantly positively associated with older age (β = 0.2; *p* < 0.01), higher educational level (β = 0.2; *p* < 0.01), higher SES (β = 0.2; *p* < 0.01), physical activity during pregnancy (β = 0.2; *p* < 0.01), and complications during pregnancy (β = 0.3; *p* = 0.04) and negatively with a single marital status (β = −0.1; *p* = 0.05), lower SES (β = −0.2; *p* = 0.03), place of residence with fewer than 10 thousands of inhabitants (β = −0.2; *p* = 0.01), and a higher level of stress (β = −0.1; *p* = 0.02). Looking at the scores’ difference (Prudent pattern score minus Western pattern score), as an overall measure of the respondents’ relative tendency towards a Prudent or Western dietary pattern, several determinants were confirmed. Older age (β = 0.2; *p* < 0.01), higher educational level (β = 0.3; *p* < 0.01) and SES (β = 0.2; *p* < 0.01), overweight/obesity before (β = 0.3; *p* < 0.01) as well as physical activity during pregnancy (β = 0.2; *p* = 0.01) were positive determinants of a healthier diet (increasing Prudent–Western dietary pattern scores). On the other hand, parity (β = −0.2; *p* = 0.04) and living in small cities (β = −0.3; *p* = 0.02) were significantly related to Western dietary pattern. Similar results were obtained based on the multiple logistic regression model (Table S4).

## 4. Discussion

In the current prospective cohort study, two distinct dietary patterns (Prudent and Western), based on the data from FFQ collected during the 2nd trimester of pregnancy, were identified. Following the multivariate regression analysis, clear sociodemographic, environmental, lifestyle, and pregnancy-related determinates of diet quality were established. Older age, higher educational level and SES, overweight/obesity before pregnancy as well as physical activity during it were significant determinants of a healthier diet while becoming pregnant. On the other hand, parity (more than one child in the family) and living in a small city were significantly related to a Western dietary pattern. These characteristics need to be accounted for in educational programs and interventions focusing on healthy diet recommendations during pregnancy. 

Considering its complexity, a diet is methodologically difficult to capture and no gold-standard method exists [11]. Dietary quality and dietary pattern approaches were most frequently used to evaluate the diet as a whole. The first one is defining a diet by scoring adherence to dietary guidelines, rating the diversity of food choice in key food groups or scoring predefined food patterns known to protect or impair health [11,27,28,29,30,31,32,33,34,35,36]. Exploratory factor analysis, as selected in the current study, and principal components analysis are two similar techniques employed to generate empirically derived dietary patterns. In that analysis, dietary data most commonly collected through FFQ or dietary records are reduced into dietary patterns based on intercorrelations among individual items [9,10,11,37,38,39,40,41,42]. Despite methodological differences such as dietary profile labeling and the varied range of foods consumed by different population groups, in most analyses (similarly to the current one), a healthy diet, with high factor loadings for whole grains, fruit, vegetables, legumes, and fish, and a less-healthy diet, with high factor loading for refined grains, processed meats, and sweets, have been described [37]. Other dietary patterns, reported in previous studies, most often reflected regional and cultural influences on dietary intake (as an example: Common Brazilian pattern: Rice/beans/pasta, French roll, margarine, boneless beef/ chicken/eggs, coffee/artificial juices, sugar in the study by Hoffmann et al. (2013) [39] or US Southern pattern: Eggs, cooked cereals, peaches, corn, fried fish, beans, greens, cabbage, sweet potatoes, liver, pig’s feet, neck bones, oxtails, tongue, pork, and real fruit juices in the study by Völgyi et al. (2013)) [41]. 

Dietary patterns are specific to particular populations. They may vary with age, socioeconomic status, ethnicity, cultural traditions, and food availability. The existing research confirms that there are important differences in dietary profiles between and within Eastern and Western European countries [43,44,45]. Thus, it is important to analyze them in different populations to develop relevant prevention strategies. 

The results of the current as well as of the other studies consistently point to a social gradient whereby women who are older, more educated, and with a higher SES are more likely to follow healthier dietary patterns [11,38,40,41]. It is worth noting that pregnancy is the period of greater motivation for a behavior change, and it is also the great potential for health interventions. Persistence of social gradient in a diet during pregnancy might indicate that women’s motivation is not enough, health promotion potential is not used sufficiently or that neither can overcome the wider social forces in play [46].

Among lifestyle-related factors, smoking status, physical activity, and pre-pregnancy BMI were the most frequently evaluated [11]. Other aspects of health behavior, including supplement use, weight gain, alcohol drinking, caffeine, and stress/depression/anxiety, were less commonly evaluated [27,30,32,34,38,46,47]. Similarly to other studies, we observed that the women who declared leisure-time physical activity during pregnancy represented healthier dietary choices [11,27,31,38,42]. Although we did not observe a statistically significant impact of alcohol and smoking on a dietary pattern during pregnancy, our results are in a similar direction to those noted by other research, namely that alcohol consumption and active smoking status are determinants of unhealthy dietary patterns [11,28,38,40]. 

In the REPRO_PL cohort, women who used supplements during pregnancy did not significantly differ in respect to their dietary choices from those who did not. Similar results were observed by Fowler et al. (2012) [47]. By contrast, Laraia et al. (2007) indicated that women who had used any vitamins before pregnancy had a significantly higher diet quality index [30]. It needs to be underlined that 92% of the women in our cohort used supplements/vitamins at the beginning of their pregnancy as it is highly recommended by gynecologists and obstetricians. That can be responsible for the lack of an association. 

In our assessment, we evaluated the level of stress (PSS measuring disturbances of daily hassles) as a potential determinant of a maternal diet during pregnancy. However, the results were not statistically significant. It is worth noting that in our cohort, the women declared a generally medium level of stress (median 17, range 0–38 points), which, in addition to other variables which are more important for dietary choices, can be the reason for the lack of an association.

The relationship between pre-pregnancy BMI and dietary pattern in pregnancy is less consistent across the existing studies and more difficult to interpret [11]. Unexpectedly, in our assessment, we found that the women who were overweight/obese before pregnancy indicated a healthier dietary pattern during the pregnancy period. In that perspective, it could be suspected that they changed their nutritional behavior in the entire pregnancy (for their and newborn’s health). That is supported by the observation that the mean energy intake during pregnancy among the women classified as overweight/obese did not significantly differ from that among the women with pre-pregnancy BMI within the recommended range (1572.12 ± 382.29 kcal. vs. 1619.54 ± 362.87 kcal. *p* = 0.3) (data not shown). In addition, weight gain during pregnancy was not identified as a significant determinant of dietary patterns (*p* > 0.05). However, underreporting cannot be excluded. Women who are overweight/obese often experience weight stigma, and their higher body mass indexes frequently correspond to higher underreporting and poorer diet quality [48,49,50,51].

Environmental and pregnancy-related factors other than parity were less commonly investigated [11]. Our analysis indicated that the women living in the cities/villages with fewer than 10 thousand inhabitants were less willing to follow healthy dietary patterns. This can be related to a few factors, including limited access to varied food resources, as they could more likely follow more traditional dietary patterns or patterns associated with local products; moreover, women living in small villages might have more limited access to preventive measures, such as educational programs and support for a healthy diet in pregnancy. Previous studies have found that women living in urban areas had increased odds of low dietary quality or of having a low healthy eating index [35,46]. This can indicate that such associations might be country- or local-specific. Similarly to the results from the study by Northstone et al. (2008), in our assessment, the women who completed the questionnaire in the summer months (June–August) had slightly higher, although not statistically significant, scores for a healthier dietary pattern than those who did this in the winter season (December–February) [38]. 

Multiparity was significantly related to a rather Western dietary pattern in our cohort. To date, findings on parity have been inconsistent [11,38,40,41,52]. It is possible that this is due to confounding, i.e., parity acting as a marker of sociodemographic factors (age, marital status, education, SES, and number of family members in a household) or that the influence of parity is context-specific, e.g., differences in resources and support allocated to the women in their first pregnancy and women who already have children [11]. Other pregnancy-related factors, such as week of pregnancy for the 1st prenatal visit (as the proxy of women’s awareness, health status, as well as potential for lifestyle-related advice) and pregnancy complications were not significantly related to dietary patterns in our analysis. This might result from inaccurately stated measures. Number of medical-care visits and nausea (measured separately from other symptoms and complications) and food aversions could be more reliable for this assessment. 

A strength of the current study is related to the prospective study design, which allows for a reliable assessment of the potential determinants and dietary-related variables, with some of them being assessed by a nurse, gynecologist/obstetrician (including pregnancy complications that appear after inclusion into the study, maternal height, supplement use) or by a biomarker measurement (cotinine level in saliva for verification of smoking status). It is also worth mentioning the study inclusion criteria, i.e., healthy women (without diseases as specified by the study protocol, such as diabetes mellitus, hypertension), in healthy pregnancy not assisted with reproduction procedures, which in principle means that women not requiring special diets. Women were excluded from the analysis if they described themselves as vegetarian/vegan or as following specific diet restrictions. In addition, according to the conclusions from a systematic review of the results from the studies focusing on determinants of dietary patterns and diet quality during pregnancy published by Doyle et al. (2016), we focused not only on the sociodemographic variables (5 factors) but additionally on environmental (2 factors), lifestyle (7 factors), and pregnancy-related factors (3 factors) [11]. That might give a more accurate assessment of diet predictors.

Limitations of the study also need to be mentioned. Firstly, the factor analysis is based on subjective decisions, and the obtained results can be influenced by choice of factor loading cut-offs and rotation methods [9,10,11,37]. In addition, as mentioned previously, dietary patterns differ between places, populations, and cultural contexts, so a direct comparison might be difficult. However, in our population, we identified the two most frequently reported patterns (healthy and processed pattern), and the obtained results are similar to those published in other studies. Finally, although 17 potential determinants were evaluated, taking into account the study design and methods (not only limited to a diet and its determinants), we did not cover food aversions as well as social capital and support, food policies, and recommendations (by a physician or at a country level) or shape concern, which all can impact women’s decisions regarding dietary choices. 

## 5. Conclusions

Appropriate and balanced nutrition during pregnancy is one of the key elements of a healthy lifestyle to optimize both maternal and children’s health. Results from the current assessment as well as other studies in this field have underlined some sociodemographic (age, educational level, SES), lifestyle (physical activity, smoking status), environmental (place of residence), and pregnancy-related (parity) determinants of maternal dietary patterns during pregnancy, suggesting that extra resources may be necessary, especially for disadvantaged mothers, to ensure healthy nutritional choices. Different approaches to promote behavioral change and more targeted nutrition programs (at a national and local level) may be required to improve nutritional status of pregnant women.

## Figures and Tables

**Table 1:** Factor loadings of the food groups in dietary patterns (N = 1306) *.

Food Groups	Prudent	Western
Refined grains	0.06	**0.57**
Whole grains	**0.33**	**−0.53**
Low–fat dairy	**0.38**	−0.13
High–fat dairy	**0.29**	0.04
Butter	0.06	**0.28**
Red meat	**0.29**	0.15
Poultry	**0.38**	0.15
Processed meat	**0.22**	**0.31**
Fish/Seafood	**0.28**	−0.10
Fruits	**0.44**	0.05
Vegetables	**0.50**	−0.03
Potatoes	**0.24**	**0.41**
Legumes	**0.37**	−0.07
Sweets	**0.23**	**0.29**
Variance explained (%)	9.8	7.9

* Food groups with bold numbers are considered to have an interpretable association (absolute value of factor loading ≥0.2) with a factor.

**Table 2:** Sociodemographic, lifestyle, environmental, and pregnancy-related factors and dietary patterns during pregnancy (N = 1306) *.

Variables	Western	Mixed	Prudent	*p*
**Sociodemographic**				
**Maternal age (years)**				0.05
17–30	296 (35.4)	279 (33.3)	262 (31.3)	
>30	130 (29.1)	153 (34.3)	163 (36.5)	
**Marital status**				0.03
Married	333 (31.9)	351 (33.6)	361 (34.5)	
Unmarried	97 (38.8)	87 (34.8)	66 (26.4)	
**Maternal education (years of education)**				<0.01
≤9	17 (56.7)	4 (13.3)	9 (30)	
10–12	153 (42.4)	130 (36)	78 (21.6)	
>12	261 (28.9)	303 (33.5)	340 (37.6)	
**Occupational activity between the 8th-12th week of pregnancy**				0.03
No	178 (34.8)	187 (36.6)	146 (28.6)	
Yes	231 (31.9)	233 (32.2)	260 (35.9)	
**Socio-economic status**				<0.01
Low	40 (42.1)	32 (33.7)	23 (24.2)	
Medium	296 (34.1)	300 (34.6)	271 (31.3)	
High	90 (28.6)	97 (30.8)	128 (40.6)	
**Lifestyle/health behavior**				
**Pre-pregnancy BMI (kg/m^2^)**				<0.01
<18.5	47 (41.2)	40 (35.1)	27 (23.7)	
18.5–24.99	327 (35)	298 (31.9)	308 (33)	
≥25	54 (22.6)	96 (40.2)	89 (37.2)	
**Gestational weight gain**				0.29
Recommended	136 (32)	145 (34.1)	144 (33.9)	
Low	177 (31.9)	149 (35)	183 (33)	
High	88 (39.3)	74 (33)	62 (27.7)	
**Supplement use**				0.04
No	22 (26.2)	39 (46.4)	23 (27.4)	
Yes	407 (33.8)	395 (32.8)	401 (33.3)	
**Cotinine level**				<0.01
≤10ng/ml	368 (31.8)	394 (34)	396 (34.2)	
>10ng/ml	67 (46.2)	44 (30.3)	34 (23.4)	
**Alcohol consumption**				0.56
No	397 (32.9)	407 (33.7)	403 (33.4)	
Yes	29 (34.9)	31 (37.3)	23 (27.7)	
**Physical activity**				<0.01
No	161 (39.7)	129 (31.8)	116 (28.6)	
Yes	274 (30.5)	310 (34.6)	313 (34.9)	
**Perceived Stress Scale (range: 0–38 points)**				0.11
<17 points	192 (31.2)	204 (33.2)	219 (35.6)	
≥17 points	243 (35.6)	231 (33.9)	208 (30.5)	
**Environmental**				
**Place of residence (thousands of inhabitants)**				<0.01
<10	115 (42.9)	85 (31.7)	68 (25.4)	
10–100	65 (33.5)	62 (32)	67 (34.5)	
100–500	102 (29)	128 (36.4)	122 (34.7)	
>500	153 (31.2)	165 (33.7)	172 (35.1)	
**Season**				0.62
December–February	106 (34)	11 (35.6)	95 (30.4)	
March–May	111 (34)	107 (32.8)	108 (33.1)	
June–August	90 (33.5)	85 (31.6)	94 (34.9)	
September–November	86 (33.6)	98 (38.3)	72 (28.1)	
**Pregnancy-related**				
**Parity**				0.23
0	212 (31.4)	228 (33.8)	235 (34.8)	
≥1	220 (35.3)	211 (33.8)	193 (30.9)	
**Pregnancy symptoms and complications**				0.37
No	422 (33.5)	428 (33.9)	411 (32.6)	
Yes	14 (31.1)	12 (26.7)	19 (42.2)	
**Week of pregnancy of the 1st medical-care visit**				0.16
≤6	242 (31.1)	270 (34.7)	267 (34.3)	
>6	183 (36.2)	164 (32.4)	159 (31.4)	
**Sex of the child**				0.13
Male	184 (31.6)	214 (36.8)	184 (31.6)	
Female	196 (34.5)	177 (31.2)	195 (34.3)	

* Dietary pattern score: Prudent pattern score minus Western pattern score difference was categorized into three categories (by two cut-off points: 33.3rd and 66.6th percentiles): Western pattern [−4.25, −1.37], N = 436 (33.4%); Mixed pattern (−1.37, −0.44], N = 440 (33.7%); Prudent pattern (−0.44, 4.42], N = 430 (32.9%).

**Table 3:** Sociodemographic, lifestyle, environmental, and pregnancy-related determinants of dietary pattern scores during pregnancy—multivariate linear regression model (N = 958) *.

Variables	Western	Prudent	Prudent-Western
	β (95%CI) *p*		
**Sociodemographic**			
**Maternal age (years)**			
17–30	Ref.	Ref.	Ref.
>30	−0.08 (−0.21, 0.05) 0.25	0.16 (0.04, 0.27) 0.007	0.24 (0.08, 0.40) 0.004
**Marital status**			
married	Ref.	Ref.	Ref.
unmarried	0.03 (−0.13, 0.20) 0.68	−0.13 (−0.27, 0.00) 0.05	−0.17 (−0.36, 0.02) 0.09
**Maternal education (years of education)**			
10–12	Ref.	Ref.	Ref.
≤9	0.24 (−0.26, 0.75) 0.34	−0.04 (−0.46, 0.38) 0.84	−0.29 (−0.88, 0.31) 0.34
>12	−0.10 (−0.24, 0.04) 0.16	0.16 (0.04, 0.28) 0.007	0.26 (0.10, 0.43) 0.002
**Occupational activity between the 8th-12th week of pregnancy**			
No	Ref.	Ref.	Ref.
Yes	−0.11 (−0.23, 0.02) 0.09	−0.05 (−0.16, 0.05) 0.32	0.05 (−0.09, 0.20) 0.46
**Socioeconomic status**			
Medium	Ref.	Ref.	Ref.
Low	−0.08 (−0.32, 0.15) 0.49	−0.23 (−0.43, −0.03) 0.03	−0.15 (−0.43, 0.14) 0.31
High	−0.07 (−0.21, 0.06) 0.29	0.17 (0.05, 0.28) 0.005	0.24 (0.08, 0.40) 0.004
**Lifestyle/health behavior**			
**Pre-pregnancy BMI**			
18.5–24.99	Ref.	Ref.	Ref.
<18.5	0.02 (−0.20, 0.24) 0.89	−0.10 (−0.28, 0.09) 0.29	−0.12 (−0.38, 0.15) 0.38
≥25	−0.30 (−0.46, −0.14) <0.001	0.03 (−0.10, 0.17) 0.61	0.33 (0.14, 0.52) 0.001
**Gestational weight gain**			
Recommended	Ref.	Ref.	Ref.
Low	−0.07(−0.20, 0.06) 0.31	0.00 (−0.11, 0.11) 0.99	0.07 (−0.09, 0.22) 0.39
High	0.12 (−0.05, 0.29) 0.17	−0.01 (−0.15, 0.14) 0.92	−0.13 (−0.33, 0.08) 0.22
**Supplement use**			
No	Ref.	Ref.	Ref.
Yes	0.04 (−0.21, 0.28) 0.78	0.01 (−0.20, 0.22) 0.95	−0.03 (−0.32, 0.27) 0.85
**Cotinine level**			
≤10ng/ml	Ref.	Ref.	Ref.
>10ng/ml	0.13 (−0.06, 0.33) 0.17	−0.04 (−0.20, 0.13) 0.66	−0.17 (−0.40, 0.06) 0.15
**Alcohol consumption**			
No	Ref.	Ref.	Ref.
Yes	0.16 (−0.07, 0.39) 0.16	0.03 (−0.16, 0.22) 0.76	−0.13 (−0.41, 0.14) 0.34
**Physical activity**			
No	Ref.	Ref.	Ref.
Yes	−0.03 (−0.15, 0.10) 0.67	0.16 (0.06, 0.27) 0.003	0.19 (0.04, 0.34) 0.01
**Perceived Stress Scale (range 0–38)**			
<17 points	Ref.	Ref.	Ref.
≥17 points	−0.07 (−0.19, 0.04) 0.22	−0.12 (−0.22, −0.02) 0.02	−0.04 (−0.18, 0.10) 0.56
**Environmental**			
**Place of residence**			
>500	Ref.	Ref.	Ref.
<10	0.07 (−0.10, 0.24) 0.43	−0.18 (−0.32, −0.04) 0.01	−0.25 (−0.44, −0.05) 0.02
10–100	0.08 (−0.11, 0.26) 0.41	−0.03 (−0.18, 0.13) 0.73	−0.10 (−0.32, 0.11) 0.35
100–500	0.02 (−0.13, 0.17) 0.82	0.04 (−0.09, 0.16) 0.53	0.02 (−0.15, 0.20) 0.80
**Season**			
December–February	Ref.	Ref.	Ref.
March–May	0.01 (−0.14, 0.17) 0.89	0.01 (−0.12, 0.14) 0.84	0.00 (−0.18, 0.19) 0.98
June–August	−0.13 (−0.30, 0.03) 0.11	0.02 (−0.12, 0.16) 0.79	0.15 (−0.04, 0.35) 0.13
September–November	−0.07 (−0.24, 0.09) 0.40	−0.06 (−0.20, 0.08) 0.37	0.01 (−0.19, 0.20) 0.94
**Pregnancy-related**			
**Parity**			
0	Ref.	Ref.	Ref.
≥1	0.26 (0.13, 0.39) <0.001	0.10 (−0.01, 0.21) 0.07	−0.16 (−0.32, −0.01) 0.04
**Pregnancy symptoms and complications**			
No	Ref.	Ref.	Ref.
Yes	0.26 (−0.06, 0.59) 0.11	0.29 (0.02, 0.57) 0.04	0.03 (−0.36, 0.41) 0.89
**Week of pregnancy of the 1st medical-care visit**			
≤6	Ref.	Ref.	Ref.
>6	0.01 (−0.11, 0.13) 0.91	0.03 (−0.07, 0.13) 0.56	0.02 (−0.12, 0.17) 0.75
**Sex of the child**			
Male	Ref.	Ref.	Ref.
Female	−0.05 (−0.17, 0.07) 0.41	−0.02 (−0.12, 0.08) 0.69	0.03 (−0.11, 0.17) 0.68

* Dietary patterns during pregnancy: Prudent pattern score, Western pattern score, and their scores’ difference: Prudent pattern score minus Western pattern score; Ref.: Reference group.

## Supplementary Materials

The following are available online https://www.mdpi.com/1660-4601/16/5/754/s1, Table S1. Characteristics of the study population (N = 1306); Table S2. Frequency of consumption of the grouped food products; Table S3. Seasonality of consumption of the grouped food products; Table S4. Sociodemographic, lifestyle, environmental, and pregnancy-related determinants of dietary pattern scores during pregnancy—multivariate logistic regression model (N = 958); Figure S1. Scree plot of 10 largest principal components for grouped FFQ scores, indicating that the first two components capture much more of the variance than any of the remaining ones.
