# Supplementary material for: Sociodemographic, Lifestyle, Environmental and Pregnancy-Related Determinants of Dietary Patterns during Pregnancy
Source: Int J Environ Res Public Health. 2019 Mar 2;16(5):754. doi: 10.3390/ijerph16050754 (PMC6427254; doi:10.3390/ijerph16050754)
Supplement: Supplementary file 1 [file ijerph-16-00754-s001.pdf]

International Journal of
Environmental Research and Public Health

MDPI

Supplementary Materials: Sociodemographic, Lifestyle, Environmental and Pregnancy-Related Determinants of Dietary Patterns during Pregnancy

Table S1. Characteristics of the study population (N = 1306).

Variables	Category	N (%)
**Sociodemographic**		
**Maternal age (years)**		
	17-30	837 (64.1)
	>30	446 (34.2)
	No data	23 (1.8)
**Marital status**		
	Married	1045 (80.0)
	Unmarried	250 (19.1)
	No data	11 (0.8)
**Maternal education (years of education)**		
	≤9	30 (2.3)
	10-12	361 (27.6)
	>12	904 (69.2)
	No data	11 (0.8)
**Occupational activity between 8th-12th weeks of pregnancy**		
	No	511 (39.1)
	Yes	724 (55.4)
	No data	71 (5.4)
**Socio-economic status**		
	Low	95 (7.3)
	Medium	867 (66.4)
	High	315 (24.1)
	No data	29 (2.2)
**Lifestyle/health behavior**		
**Pre-pregnancy BMI (kg/m^2)**		
	<18.5	114 (8.7)
	18.5-24.99	933 (71.4)
	≥25	239 (18.3)
	No data	20 (1.5)
**Cotinine level**		
	≤10ng/ml	1158 (88.7)
	>10ng/ml	145 (11.1)
	No data	3 (0.2)
**Alcohol consumption**		
	No	1207 (92.4)
	Yes	83 (6.4)
	No data	16 (1.2)
**Physical activity**		
	No	406 (31.1)
	Yes	897 (68.7)
	No data	3 (0.2)
**Perceived Stress Scale (range 0-38 points)**		
	<17 points	615 (47.1)
	≥ 17 points	682 (52.2)
	No data	9 (0.7)
**Environmental**		
**Place of residence (thousands of inhabitants)**		
	<10	268 (20.5)

	10-100	194 (14.9)
	100-500	352 (27.0)
	>500	490 (37.5)
	No data	2 (0.2)
**Season**		
	December-February	312 (23.9)
	March-May	326 (25.0)
	June-August	269 (20.6)
	September-November	256 (19.6)
	No data	143 (10.9)
**Pregnancy-related**		
**Parity**		
	0	675 (51.7)
	≥1	624 (47.8)
	No data	7 (0.5)
**Pregnancy symptoms and complications**		
	No	1261 (96.6)
	Yes	45 (3.4)
**Week of pregnancy for the 1st medical-care visit**		
	≤6	779 (59.6)
	>6	506 (38.7)
	No data	21 (1.6)
**Sex of the child**		
	Male	582 (44.6)
	Female	568 (43.5)
	No data	156 (11.9)

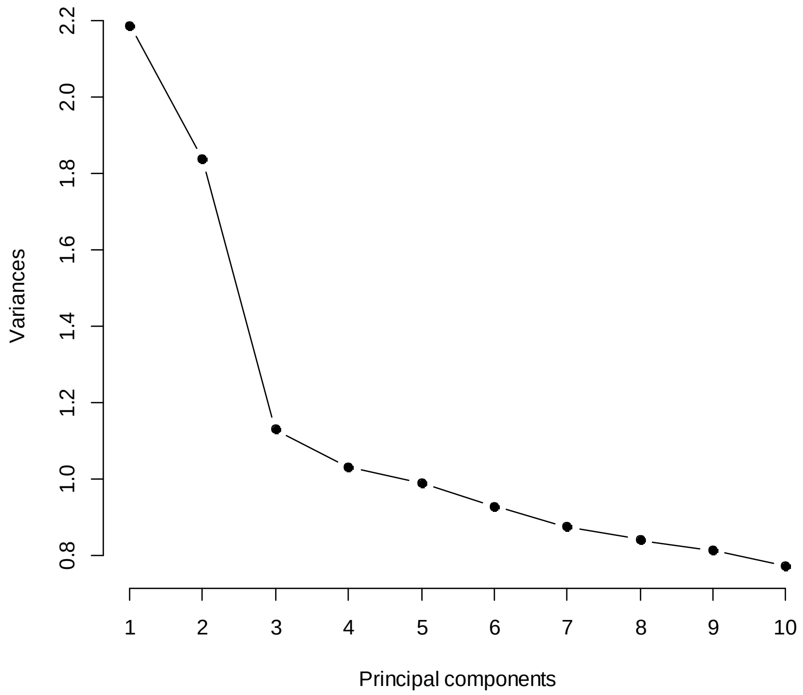

Figure S1. Scree plot of 10 largest principal components for grouped FFQ scores, indicating that the first two components capture much more of the variance than any of the remaining ones. .

Table S2. Frequency of the consumption of grouped food products.

Food Groups	Never	Less Than Once Per Month	1-3 Times Per Month	1-3 Times Per Week	4-6 Times Per Week	Everyday
	n (%)					
Refined grains	1 (0.1)	1 (0.1)	17 (1.3)	323 (24.7)	932 (71.4)	32 (2.5)
Whole grains	59 (4.5)	55 (4.2)	270 (20.7)	528 (40.4)	362 (27.7)	32 (2.5)
Low–fat dairy	1 (0.1)	0 (0)	19 (1.5)	403 (30.9)	807 (61.8)	76 (5.8)
High–fat dairy	62 (4.7)	18 (1.4)	141 (10.8)	623 (47.7)	308 (23.6)	154 (11.8)
Butter	35 (2.7)	8 (0.6)	22 (1.7)	144 (11)	500 (38.3)	597 (45.7)
Red meat	73 (5.6)	45 (3.4)	288 (22.1)	676 (51.8)	204 (15.6)	20 (1.5)
Poultry	41 (3.1)	9 (0.7)	180 (13.8)	859 (65.8)	197 (15.1)	20 (1.5)
Processed meat	47 (3.6)	16 (1.2)	54 (4.1)	383 (29.3)	445 (34.1)	361 (27.6)
Fish/Seafood	70 (5.4)	126 (9.6)	670 (51.3)	421 (32.2)	18 (1.4)	1 (0.1)
Fruits	0 (0)	0 (0)	7 (0.5)	482 (36.9)	774 (59.3)	43 (3.3)
Vegetables	3 (0.2)	1 (0.1)	25 (1.9)	611 (46.8)	650 (49.8)	16 (1.2)
Potatoes	15 (1.1)	15 (1.1)	97 (7.4)	606 (46.4)	438 (33.5)	135 (10.3)
Legumes	193 (14.8)	152 (11.6)	467 (35.8)	374 (28.6)	113 (8.7)	7 (0.5)
Sweets	45 (3.4)	42 (3.2)	260 (19.9)	656 (50.2)	258 (19.8)	45 (3.4)

Table S3. Seasonality of the consumption of grouped food products.

Food Groups	*p*
Refined grains	0.56
Whole grains	0.21
Low–fat dairy	0.49
High–fat dairy	0.99
Butter	0.75
Red meat	0.51
Poultry	0.84
Processed meat	0.47
Fish/Seafood	0.35
Fruits	**0.001**
Vegetables	**0.006**
Potatoes	0.41
Legumes	0.97
Sweets	0.73

Table S4. Sociodemographic, lifestyle, environmental and pregnancy-related determinants of dietary pattern scores during pregnancy* – multivariate logistic regression model (n = 958).

	Western		Prudent		Prudent -Western	
**Variables**	**OR (95%CI)**	***p***	**OR (95%CI)**	***p***	**OR (95%CI)**	***p***
**Sociodemographic**						
**Maternal age at delivery (years)**						
17-30	Ref.		Ref.		Ref.	
>30	1.00 (0.74, 1.36)	0.99	1.54 (1.13, 2.10)	**0.006**	1.39 (1.02, 1.89)	**0.04**
**Marital status**						
Married	Ref.		Ref.		Ref.	
Unmarried	1.19 (0.82, 1.72)	0.37	0.83 (0.57, 1.21)	0.34	0.70 (0.48, 1.02)	0.06
**Maternal education (years of education)**	Ref.		Ref.		Ref.	
10-12						
≤9	2.68 (0.84, 10.36)	0.11	0.74 (0.19, 2.41)	0.63	0.64 (0.16, 2.05)	0.47
>12	0.91 (0.67, 1.25)	0.57	1.60 (1.16, 2.21)	**0.004**	1.59 (1.15, 2.20)	**0.005**
**Occupational activity between 8th-12th weeks of pregnancy**						
No	Ref.		Ref.		Ref.	
Yes	0.86 (0.65, 1.14)	0.29	0.84 (0.63, 1.11)	0.22	0.98 (0.74, 1.30)	0.91
**Socio – economic status**						
Medium	Ref.		Ref.		Ref.	
Low	0.70 (0.41, 1.21)	0.21	0.47 (0.26, 0.83)	**0.011**	0.90 (0.51, 1.57)	0.71
High	0.92 (0.67, 1.26)	0.60	1.54 (1.12, 2.12)	**0.008**	1.34 (0.98, 1.84)	0.07
**Lifestyle/health behavior**						
**Pre-pregnancy BMI**						
18.5-24.99	Ref.		Ref.		Ref.	
<18.5	1.16 (0.72, 1.91)	0.54	0.68 (0.41, 1.11)	0.12	0.84 (0.51, 1.38)	0.49

≥25	0.65 (0.46, 0.92)	**0.02**	1.13 (0.79, 1.61)	0.50	1.57 (1.11, 2.24)	**0.01**
**Cotinine level**						
≤10ng/ml	Ref.		Ref.		Ref.	
>10ng/ml	1.55 (1.00, 2.45)	**0.054**	0.92 (0.59, 1.46)	0.74	0.79 (0.50, 1.23)	0.29
**Alcohol consumption**						
No	Ref.		Ref.		Ref.	
Yes	1.78 (1.05, 3.05)	**0.03**	1.08 (0.63, 1.83)	0.78	0.65 (0.38, 1.10)	0.11
**Physical activity**						
No	Ref.		Ref.		Ref.	
Yes	0.82 (0.61, 1.09)	0.17	1.30 (0.97, 1.73)	0.08	1.50 (1.12, 2.00)	**0.007**
**Perceived Stress Scale (range0-38)**						
<17	Ref.		Ref.		Ref.	
≥ 17	0.91 (0.69, 1.19)	0.47	0.83 (0.63, 1.10)	0.19	0.88 (0.67, 1.15)	0.34
**Environmental**						
**Place of residence (thousands of inhabitants)**	Ref.		Ref.		Ref.	
>500	1.21 (0.83, 1.77)	0.32	0.64 (0.44, 0.95)	**0.03**	0.74 (0.50, 1.08)	0.12
<10						
10-100	1.24 (0.82, 1.89)	0.31	0.94 (0.61, 1.43)	0.76	0.96 (0.63, 1.46)	0.84
100-500	1.12 (0.80, 1.57)	0.52	1.10 (0.78, 1.54)	0.59	1.12 (0.80, 1.57)	0.52
**Season**						
December-February	Ref.		Ref.		Ref.	
March-May	1.03 (0.72, 1.46)	0.89	1.24 (0.87, 1.77)	0.24	0.77 (0.54, 1.10)	0.15
June-August	0.67 (0.46, 0.98)	**0.04**	1.37 (0.93, 2.02)	0.11	1.16 (0.79, 1.70)	0.44
September-November	0.71 (0.49, 1.04)	0.08	0.96 (0.65, 1.40)	0.83	1.02 (0.70, 1.50)	0.89
**Pregnancy-related**						
**Parity**						
0	Ref.		Ref.		Ref.	
≥1	1.54 (1.15, 2.08)	**0.004**	1.20 (0.89, 1.62)	0.23	0.64 (0.47, 0.87)	**0.004**
**Pregnancy symptoms and complications**	Ref.		Ref.		Ref.	
No	1.67 (0.80, 3.63)	0.18	1.67 (0.78, 3.74)	0.19	1.47 (0.70, 3.16)	0.31
Yes						
**Week of pregnancy for the first medical care visit**	Ref.		Ref.		Ref.	
≤6	1.00 (0.76, 1.31)	0.99	1.07 (0.81, 1.41)	0.64	1.09 (0.83, 1.44)	0.53
>6						
**Sex of the child**						
Male	Ref.		Ref.		Ref.	
Female	0.96 (0.74, 1.25)	0.77	0.79 (0.61, 1.04)	0.09	1.01 (0.77, 1.32)	0.95

*Logistic regression modeling for Western dietary pattern, Prudent dietary pattern and their scores' difference: Prudent pattern score minus Western pattern score being over their respective median values. OR – odds ratio. 95%CI – 95% confidence interval. Ref. – reference group.
